# Determination of Non-Recrystallization Temperature for Niobium Microalloyed Steel

**DOI:** 10.3390/ma14102639

**Published:** 2021-05-18

**Authors:** Mohammad Nishat Akhtar, Muneer Khan, Sher Afghan Khan, Asif Afzal, Ram Subbiah, Sheikh Nazir Ahmad, Murtuja Husain, Mohammad Mursaleen Butt, Abdul Rahim Othman, Elmi Abu Bakar

**Affiliations:** 1 School of Aerospace Engineering, Universiti Sains Malaysia, Nibong Tebal 14300, Malaysia; nishat@usm.my; 2 Department of Mechanical Engineering, NIT, Srinagar, Jammu and Kashmir 190006, India; muneerkhan680946@gmail.com (M.K.); nazir@nitsri.net (S.N.A.); mursaleen@nitsri.net (M.M.B.); 3 Department of Mechanical Engineering, Faculty of Engineering, IIUM, Gombak Campus, Kuala Lumpur 53100, Malaysia; sakhan@iium.edu.my; 4 Department of Mechanical Engineering, P. A. College of Engineering (Affiliated to Visvesvaraya Technological University, Belagavi), Mangaluru 574153, India; 5 Department of Mechanical Engineering, Gokaraju Rangaraju Institute of Engineering & Technology, Hyderabad, Telangana 500090, India; ram4msrm@gmail.com; 6 CSIR-National Metallurgical Laboratory, Jamshedpur 831001, India; murtaja02bit@gmail.com; 7 Department of Mechanical Engineering, Universiti Teknologi PETRONAS, Seri Iskandar 32610, Malaysia

**Keywords:** microalloy, niobium, recrystallization temperature, microstructure, temperature

## Abstract

In the present investigation, the non-recrystallization temperature (T_NR_) of niobium-microalloyed steel is determined to plan rolling schedules for obtaining the desired properties of steel. The value of T_NR_ is based on both alloying elements and deformation parameters. In the literature, T_NR_ equations have been developed and utilized. However, each equation has certain limitations which constrain its applicability. This study was completed using laboratory-grade low-carbon Nb-microalloyed steels designed to meet the API X-70 specification. Nb- microalloyed steel is processed by the melting and casting process, and the composition is found by optical emission spectroscopy (OES). Multiple-hit deformation tests were carried out on a Gleeble^®^ 3500 system in the standard pocket-jaw configuration to determine T_NR_. Cuboidal specimens (10 (L) × 20 (W) × 20 (T) mm^3^) were taken for compression test (multiple-hit deformation tests) in gleeble. Microstructure evolutions were carried out by using OM (optical microscopy) and SEM (scanning electron microscopy). The value of T_NR_ determined for 0.1 wt.% niobium bearing microalloyed steel is ~ 951 °C. Nb- microalloyed steel rolled at T_NR_ produce partially recrystallized grain with ferrite nucleation. Hence, to verify the TNR value, a rolling process is applied with the finishing rolling temperature near TNR (~951 °C). The microstructure is also revealed in the pancake shape, which confirms T_NR_.

## 1. Introduction

Microalloyed (MA) steels have become increasingly popular, particularly in the plate and pipeline steel applications where larger diameter pipes are being developed with the need for increased strength, formability, and joinability [1,2]. In combination with processing parameters, small additions of V (vanadium), Nb (niobium), and Ti (titanium) to HSLA (high strength low alloy) steels are designed to achieve higher strength while minimizing required plate thicknesses for the desired application. Microalloying additions are generally used for grain refinement, to influence recrystallization behavior, and for precipitation strengthening through the formation of carbides or nitrides [3,4]. The MA steels for pipeline needs a well-planned rolling schedule, which depends upon a critical temperature known as non-recrystallization temperature (T_NR_) to obtain partially crystallized grains. 

Bauer et al. [5] discussed that the most common mechanical properties required for optimum pipeline performance are high strength and high toughness. Sophisticated processing routes as TMCP (thermomechanical controlled processing) allow achieving the skelp’s desired property levels by optimizing the final microstructure and crystallographic texture [5]. The steel’s critical parameters to obtain the anticipated mechanical properties are the final crystallographic texture and microstructure. The crystallographic texture, grain shape, and grain size distribution of a finished skelp are the result of a sequence of events occurring during the thermomechanical processing of the steel [4].

Chemically, the Nb is added to the pipeline steel to produce niobium carbide and carbonitride precipitation in the austenite region, which retards the austenite’s recrystallization as a result of increasing the austenite non-recrystallization temperature (T_NR_). However, during the reheating state, the austenite grain growth should be controlled to avoid possible austenite grain coarsening. For that purpose, titanium (T_i_) is added to steel [2,6] and this is also evident from the previous model developed by Khalaj et al. [7] where they investigated the grain growth behavior under the influence of pinning carbonitrides [7]. During the finishing rolling stage, niobium carbide and carbonitride precipitation occur. These precipitates pin the austenite grain boundaries, which results in non-recrystallized pancake austenite after deformation. The fine particle dispersion increases the austenite recrystallization temperature (T_NR_), slows down the bainite formation, and gives rise to adequate grain size in the base steel and the heat-affected zones after welding of the pipe [3]. In order to increase the toughness of high-frequency electrical resistance welded microalloyed line pipe steel joints, Khalaj et al. [8] came up with a novel post-welded heat treatment (PWHT) cycle which was comprised of multiple austenitizing, normalizing, quenching and tempering steps. In this regard, a comparative analysis was performed with commercial PWHT route of API X60 grade steel, which has been largely utilized for pipeline parts. Their experimental results revealed that for the proper quenching and tempering heat treatment at 600 °C for 30 min, both parameters, i.e., hardness and ultimate strength, remained unchanged related to the classic treatment [8].

Mn, Cr, Mo, Ni, or Cu elements increase the steel’s hardenability and augment the transformation from austenite to acicular ferrite and bainite. Kong et al. [4] observed that the addition of Mo’s bainite formation to the steel slowed down the transformation process. The starting and finishing temperatures were reduced, which resulted in the refinement of the bainite microstructure. It also has been observed that Cr additions result in a lower growth rate of pearlite due to the effect on retarding the C diffusivity. 

Palmiere et al. [9] observed the reheating of C-Mn steels containing Nb. They related the slab heating temperature T_RHT_ to two concepts: the temperature of dissolution (T_DISS_), i.e., the equilibrium solution temperature necessary to bring in solid solution the alloying elements and the temperature for grain coarsening (T_GC_). T_GC_ is the temperature beyond which abnormal grain coarsening or secondary recrystallization starts. The precise determination of the solid solution temperature has been the subject of many investigations dealing with a wide variety of systems [2,10,11,12,13]. Some influence of the holding time on the grain coarsening was observed by Zrnik et al. [14]. Zrnik et al. [14] found that the average grain size rises with growing coiling temperatures. The optimization of the coiling temperature window considerably stimulated the grain refinement and precipitation strengthening.

Stallybrass et al. [15] premeditated the results of different TMCP parameters on pipeline X80 graded plates’ strength and toughness. They observed that an increase in the T_RHT_ (grain coarsening) leads to an upsurge of the yield strength and a decline of toughness. The low T_RHT_ displayed the best results in both toughness tests, the Battelle drop-weight-tear tests, and the Charpy impact testing. However, Kim and Bae [16] observed only a decrease in temperature for the ductile-to-brittle transition by increasing the T_RHT_ but no influence on the upper shelf energy absorbed by the Charpy impact samples.

After each deformation during rough rolling, the recrystallization level is responsible for crystal orientations such as rotated cube originating from the austenite recrystallization texture [17]. The finish rolling passes, between the temperature value of 1000 °C and 800 °C, take place below the austenite non-recrystallization temperature (T_NR_). The austenite deformation in this TMCP step occurs due to a delay in recrystallization amongst the rolling passes. The recrystallization delay is pertinent to precipitation promoted due to Nb’s addition to the steel [18,19]. The accumulated deformation is vital to attain the necessary grain refinement essential to improve strength and toughness. There is a large body of literature on the influence of the rolling factors on pipeline steels’ final mechanical properties [20,21].

Elwazri et al. [22] have examined the influence of the precipitation hardening after rapid cooling and aging by Cu precipitation, promoted by the deformation imposed during coiling, and how this mechanism is enhanced by lowering the coiling temperatures. The influence of the coiling temperature on the fracture was reported by Lagneborg et al. [23]. The low coiling temperatures were found to decrease separation or split on the fracture appearance of Charpy samples. High cooling rates accompany it due to the delay of the phosphorous segregations for the steels, of which the purity is not appropriate.

DeArdo et al. [24] showed that extended austenite recrystallization could produce roughly ferrite grains. The grain produced through Nb (C, N) pinning grain boundaries providing more nucleation site for fine ferrite to form, as small as four μm. In contrast, Nb(C, N) precipitates are effective in retarding austenite recrystallization, thereby establishing long, flat grains with increased nucleation sites for ferrite formation. Vervynckt et al. [25] indicated that strain-induced Nb(C, N) precipitates may be about 20 nm and are large enough to subsidize strength escalations through precipitation hardening significantly. These low-temperature sediments are much more refined and contribute to greater strength increases. Niobium in solution, however, is also useful in retarding static recrystallization through solute drag effects [25,26]. Nonetheless, in order to predict the austenite grain size, a dedicated model was established by Fu et al. [27] which works on the relationship between the parameter value used to describe the grain boundary migration along with cooling rate, and the value calculated from the modified model. The value obtained from established model closely corresponds to the measured value with an average relative error of less than 5% [27]. 

### Empirical T_NR_ Determination and Its Shortcomings

An empirical formula to determine or estimate T_NR_ is a useful tool, especially for rolling mill metallurgists needing to design a rolling schedule to produce steel with specific properties without extensive laboratory testing. This subsection introduces T_NR_ equations from the literature and discusses the benefits and shortcomings of each equation. The composition limits are available in the literature. 

The Boratto equation [28,29,30] is well known for estimating T_NR_ as a function of alloy content given by:

(1) $$T_{\mathrm{NR}}=464C-\left(644\sqrt{\mathrm{Nb}}-6445\mathrm{Nb}\right)-\left(230\sqrt{V}-732V\right)+363\mathrm{Al}890T_{i}+-357\mathrm{Si}+887$$

where Nb, V, Ti, Al, C, and Si are the elements in wt.% of steel. However, the Boratto equation does not include N, well known for precipitation, and thus altering T_NR_, even though N is almost always present in low carbon commercial steels [30]. Zaky [31] has found discrepancies with the Boratto equation at low levels of Nb and V (0.01 and 0.10 wt.%, respectively) and high levels of C (above 0.17 wt.%). A simplified equation by Bai et al. [32] has been shown to produce reasonable T_NR_ estimates when the Boratto equation differs from experimental results. The formulation of equation by Bai et al. [32] is given by:

(2) $$T_{\mathrm{NR}}=174\log\left[\mathrm{Nb}\left(C+\frac{12}{14}N\right)\right]+1444$$

Nb and C are wt.% of steel, and N is the free N remaining after TiN precipitation. Another T_NR_ equation developed by Fletcher [33] used a database of 59 different T_NR_ values for 17 alloy sheets of steel. Stepwise regression was based on the Boratto equation, ignoring pass strain:

(3) $$T_{\mathrm{NR}}=849-349C+676\sqrt{\mathrm{Nb}}+337V\;\left(R^{2}=0.72\right)$$

where Nb, V, and, C are the elements in wt.%.

Deformation parameters are known to influence T_NR_. A strain-based model was developed by Bai et al. [32] given by:

(4) $$T_{\mathrm{NR}}={\beta e}^{-0.36\varepsilon }$$

where β is an alloy-dependent coefficient, calculated to be 1103 °C, 1088 °C, and 1078 °C for the steels studied by Bai et al. and *ε* is the strain. 

This model shows that small changes in pass strain can significantly affect the T_NR_. Fletcher [31] also developed a T_NR_ model based on pass strain and alloy content using a similar regression model as Equation (4) and is given by [33]:

(5) $$T_{\mathrm{NR}}=-310C+657\sqrt{\mathrm{Nb}}-149\sqrt{V}+683e^{-0.36\varepsilon }+203$$

where Nb, V, and C, are elements in wt.% and ε is the pass strain. The coefficient β was assumed to be 1100 °C for the initial analysis. A point of caution to Fletcher’s analysis is that the sign on the carbon term for Equations (3) and (5) is -ve. This is counterintuitive, as improved carbon is known to increase precipitation, inhibiting recrystallization and increasing the T_NR_. The empirical regression models may be useful to quickly predict the T_NR_ for the range of alloys used in the particular studies. They may not accurately predict T_NR_ for alloy ranges outside the reviews because the empirical constants and coefficients have no real physical meaning. Therefore, the present study is dedicated to the experimental determination of T_NR_ for niobium MA steels. 

In the rest of the manuscript, Section 2 elaborates on the methodological aspects of the proposed investigation pertaining to material design, Niobium microalloy steel processing, microstructure evaluation along with multiple deformation hit test and SEM verification. Section 3 discusses the results of the proposed investigation related to the microalloying effects. Finally, Section 4 presents the conclusion of the proposed study. 

## 2. Laboratory Experiment

This section discusses experimental materials and methods used to determine microalloying effects on T_NR_. The testing methodology and characterization techniques are discussed in detail in this section.

### 2.1. Material Design

Niobium microalloyed steel was processed through the melting and casting process to make a hot-rolled microalloyed plate. In the laboratory Nb-micro, alloyed steel was prepared to meet API X-70 specifications with the required composition that shows the same mechanical properties as X-70. Table 1 gives the chemical composition of the five alloys that were processed, followed by melting and casting. Two alloys are Nb-bearing steel, and two alloys are V- bearing steel, and one is without Nb and V with a fixed amount of other constituents.

The specific alloys were chosen to characterize precipitation and solute effects on T_NR_. A composition of microalloyed steel with 0.1 wt.% Nb was selected for this study because Nb has a good impact on increasing the T_NR_ value. Since Ti preferentially precipitates as TiN, the High-Ti alloys are expected to have most of the N in TiN. This will inhibit other precipitates from forming, thereby focusing on solid solution elements on TNR or the possible formation of TiC and formation of NbN; hence, Ti is kept at low wt.% to ensure the availability of N for the establishment of NbN.

### 2.2. Niobium MA Steel Processing

In the laboratory, by melting steel and adding the required amount of constituents, 30 kg of each composition was cast. The chemical structures were examined through standard procedures implemented at CSIR-NML Jamshedpur, i.e., optical emission microscopy. After the casting process, the microalloyed steel is further processed to clean and remove samples for experimental purposes. The sample with a dimension of 10 mm (L), 20 mm (W), and 20 mm (T) for gleeble test and sample with size 50 mm (L) × 40 mm (W) × 20 mm (T) for rolling simulation were removed from casted alloy.

### 2.3. Microstructure Evolution

As-received material tested for composition, a test to understand the austenite grain’s behavior based on soaking and quenching, was also performed in two different conditions. Two samples heated up to 1250 °C keep for 10 min, then air-cooled up to 950 °C, one kept at 950 °C for 10 min which is then water quenched (room temperature), and the other kept at 950 °C for 30 min then quenched in water (room temperature).

### 2.4. Multiple-Hit Deformation Test

**Figure 1:**
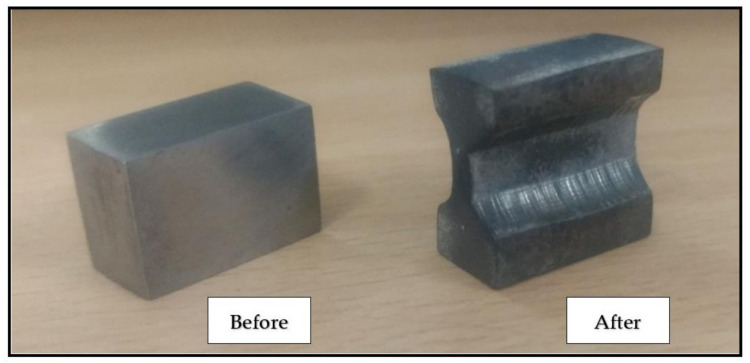
Sample before and after the compression test in gleeble.

In the present study, multiple-hit deformation experiments were performed out to estimate T_NR_ of the standard pocket-jaw set-up in the Gleeble^®^ 3500 system. Multistep hot compression tests simulate the rolling process through a series of deformation steps (i.e., hits) and continuous cooling for a given set of parameters, such as interpass time (t_ip_), rate of strain ($\dot{\varepsilon }$), strain (ε), and temperature range. The force and the amount of deformation are measured and converted into the stress–strain curve. One sample is processed for the entire temperature range from 1100 °C to 875 °C and deformed through the T_NR_. In the present investigation, the dimensions 20 (T) × 20 (W) × 10 (L) mm^3^ were used for a gleeble test in which the process parameters were: correct strain = 0.15 and 2 s^−1^ strain rates for every hit. The deformation temperatures range was 1100 °C–875 °C, with 25 °C decrements in every pass. In Figure 1, the sample before and after the greeble test is shown. The deformation parameters were as follows:

1. Heating the sample at a constant rate of 10 °C/s up to 1250 °C.
2. Soaking at the austenitizing temperature of 1250 °C for 10 min.
3. Cooling for first deformation temperature that is 1100 °C at a constant cooling rate of 10 °C/s.
4. Deformation with strain rate $\left(\dot{\varepsilon }\right)$ = 2 s^−1^ and strain (ε) = 0.15.
5. Cooling to the next deformation temperature (1075 °C) in 12.5 s at a cooling rate of 2 °C/s, the same process was repeated up 875 °C.

The above processes were repeated ten times for ten hits.

### 2.5. Determination of Non-Recrystallization (T_NR_)

**Figure 2:**
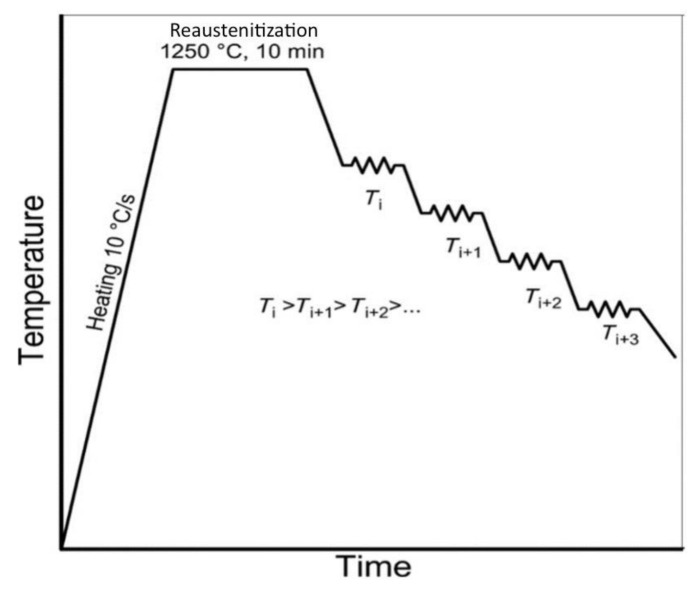
Schematic diagram of heating and cooling curve used in gleeble for compression test.

Non-recrystallization temperature (T_NR_) is determined by multiple-hit deformation tests using the mean flow stress method, as shown in Figure 2. The process is that the sample is compressed in a sudden stroke at a definite temperatures interval (25 ℃) with a fixed strain and strain rate. The time interval between every stroke is 12.5 s. During this time period the material undergoes recrystallization, which can also be called reaustenitization. After every compression sample cools down 25 ℃ for next compression, we need to find where the material stops its recrystallization process. The point where recrystallization stops material shows a sudden rise in flow stress with high slope. As the material’s recrystallization stops, it hardens and shows high resistance to the compression, hence, the high flow stress.

After every stroke the material recrystallizes form smaller austenite grains [34]. At non-recrystallization temperatures the material does not receive sufficient amounts of energy or time to complete the recrystallization process, and hence the austenite grains deform due to rolling pressure and acquire a shape like pancakes.

The value of T_NR_ can be estimated from the change of the mean flow stress (MFS) with the inverse of the absolute temperature at which the deformation takes place. In the present method, an algorithm is used to simulate the flow stress curves for multipass hot deformation processes directly from the data of a single-pass hot torsion/compression test, which needs simple equipment. As the temperature decreases consistently up to a point (finishing rolling temperature) where materials do not receive sufficient amounts of heat energy, the recrystallization process cannot be completed, hence the material start strain hardening (hardness increases rapidly), and further rolling on uncomplicated recrystallize material needs more power due high resistance to the deformation of the material. The sudden increase in deformation resistance shows high flow stress. This jump in the slope of the flow stress graph indicates the point where the recrystallization stops.

For the test to find T_NR_ point we roll down the material from 1100 ℃ to 875 ℃ at temperature intervals of 25 °C. Then, we look for the flow stress throughout the process. Here, we found that flow stress increases with small slope. However, after 951 ℃ the flow stress shows a sudden hike in the slope of flow stress graph. This point shows that now the material is hardening under the strain and a hard material shows high flow stress in rolling.

The MFS is calculated as the area under a given stress–strain curve normalized by the strain. Therefore, the MFS between strains ε_1_ and ε_2_ is calculated as follows:

(6) $$\mathrm{MFS}=\bar{\sigma }=\frac{1}{\varepsilon_{2}-\varepsilon_{1}}\int_{\varepsilon_{1}}^{\varepsilon_{2}}\sigma d\varepsilon$$

**Figure 3:**
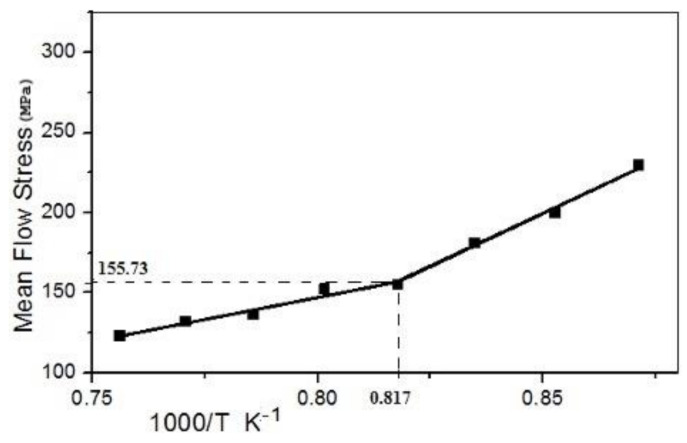
Mean flow stress curve with inverse absolute temperature K^−1^.

The MFS is determined for each pass employing Equation (6). The MFS followed this with 1000/T lines. Figure 3 shows one plot of 2 s^−1^ strain with a 12.5 s time interpass strain equal to 0.2. The properties can be separated into two ranges. The first range includes a high temperature with a lower slope with complete recrystallization. The second range has a low temperature with a moderate pitch and with a partial or no recrystallization. A crossing point, known as the T_NR_, is reached by fitting direct lines to these two segments.

### 2.6. Microstructure Verification

OM (Optical microscope) and SEM (scanning electron microscope) were used to characterize as-received and rolled material. The microstructure was evaluated in the transverse section of rolled material for received specimens as it is formed. They were analyzed transverse to the original plate rolling direction. Multiple-hit deformation was analyzed in the transverse section of the rolling movement. 

For microstructural specimens (1 cm^3^) were prepared from niobium microalloyed steel (as received). The metallographic specimens were prepared by the conventional metallographic method. The samples were etched with a 2% Nital solution. 

Three high-Nb microalloy steel samples were rolled at three different finishing temperatures, 1000 °C, 900 °C, and 800 °C, respectively. The rolled samples are further processed for microstructure evolution and confirmation of non-recrystallization temperature (T_NR_).

### 2.7. Optical Microscope and SEM Analysis

An optical microscope analyzed prior austenite grain (PAG) size and morphology. A concentrated picric acid solution was used, etching the metallographic specimen. The etchant contains a 4% saturated aqueous solution of picric acid, 2% hydrochloric acid, 2% Teepol (a wetting agent), and 92% deionized water. The etchant was heated at 65 °C–70 °C and agitated lightly. Samples were immersed for 20 s^−30^ s, rinsed with methanol, and dried with compressed air. Some samples required light back polishing to remove the appearance of martensite while retaining the PAGs.

SEM also examined the microstructure to resolve the microstructure at higher magnification. For SEM, the samples are etched by a solution containing 2% nitric acid and the rest ethanol, etched for 8 s^−10^ s.

## 3. Results and Discussion

The composition of niobium MA steel and the effects of composition are initially discussed. The physical significance of the microstructure of rolled Nb-MA steel is explained, and the determination of T_NR_ is described. 

### 3.1. Nb-MA Steel Composition

The chemical composition of fixed niobium MA steel is given in Table 2. The composition listed above has a significant effect on T_NR_ values and metal carbide and nitride formation. The steel hardenability is increased with Mn, Ni, Cr, Cu, and Mo and promotes bainite and acicular ferrite formation. V, Ti, and Nb augment the precipitation strengthening via carbide and carbonitride formation. Nb added to the MA steel composition produces carbonitride and niobium carbide in the austenite region, which retards the recrystallization of the austenite due to increasing the austenite’s non-recrystallization temperature (T_NR_). Ti is added to MA steel because its precipitates are stable up to temperatures of 1250 °C. Accordingly, these precipitates will maintain their functional ability to pin the austenite grain boundaries in the reheated slab at elevated temperatures [2]. It has been recommended that the Si and C content must be kept low to minimize the amount of the martensite–austenite constituent (M/A), which is considered to have a -ve effect on the toughness. The contents of elements such as S, P, and N contents should be reduced for general purity [9]. The presence of large TiN precipitates, promoted by high N content, together with elongated MnS inclusions and P segregations, can act as crack nucleation sites and strongly reduce the steel’s ductility. 

### 3.2. Microstructure Evolution of Heat-Treated Nb MA Steel

**Figure 4:**
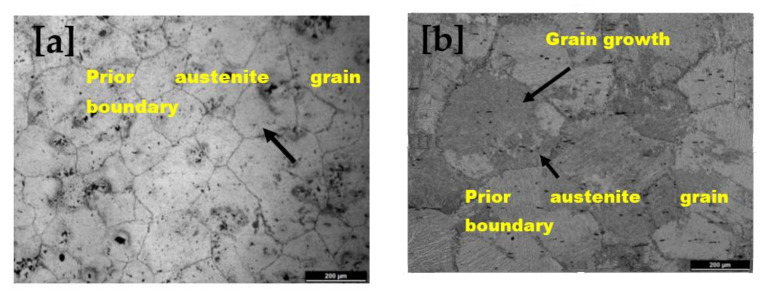
Optical micrographs showing prior austenite grains (PAG) of niobium microalloyed steel in which (**a**) heated at 1250 °C for 10 min, cooled in the air till 950 °C by holding for 10 min and then quenched at ambient temperature; (**b**) heated at 1250 °C for 10 min, cooled in the air till 950 °C by holding for 30 min and then quenched at ambient temperature.

**Figure 5:**
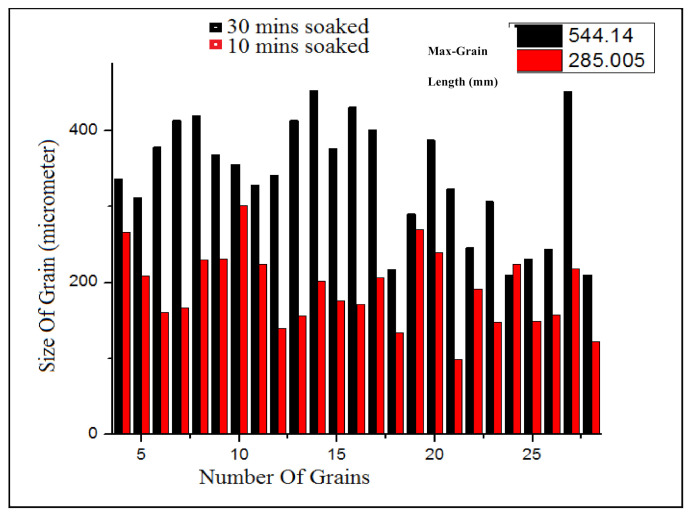
Bar graph showing the difference of grain growth with variation in the soaking time.

Optical microstructures of niobium MA steel are shown in Figure 4. Prior austenite grain boundaries of 10 min soaked can be seen in Figure 4a, while in Figure 4b, grain growth due to soaking time at 950 °C for 30 min can be noted. The grain size distribution of absorbing time 10 min and 30 min is shown in Figure 5 by the bar chart. This figure shows that a significant grain growth has occurred in the specimen with a soaking time of 30 min at 950 °C, and it might be due to soaking time at 950 °C.

### 3.3. Determination of Non-Recrystallized Temperature (T_NR_)

**Figure 6:**
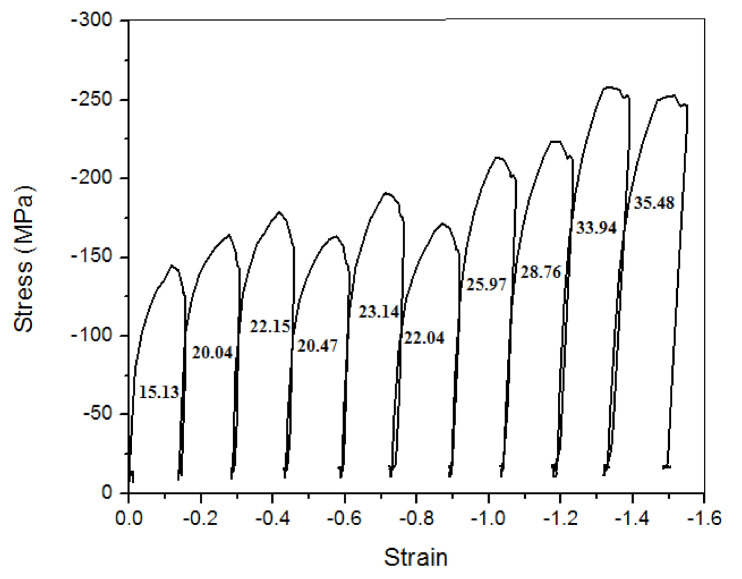
Stress-strain diagram with the area under the curve.

The stress–strain diagram of the compression test performed in the gleeble is shown in Figure 6. The calculation is performed in the area under the curve to find the mean flow stress. The given Figure 6 represents the increments in the value of stresses as the temperature decreases after every hit, and after a few hits, stress decreases from the previous value; hence, at this point, the sample becomes softest, and after this softening point, the stress starts to increase with a faster rate. 

The area under the curve is shown in Figure 6. The ratio of space under the curve to strain gives mean flow stress for that hit. 

Now the mean flow stress can be calculated by taking the ratio of area under the curve to strain, which is the same for every hit (i.e., 0.15) and is expressed as:

(7) $$\mathrm{Mean}\;\mathrm{Flow}\;\mathrm{Stress}\;(\mathrm{MFS})=\frac{Area\;under\;curve}{Strain\;in\;that\;hit}$$

All results, including the mean flow stress of compression test in gleeble, are listed in Table 3.

**Figure 7:**
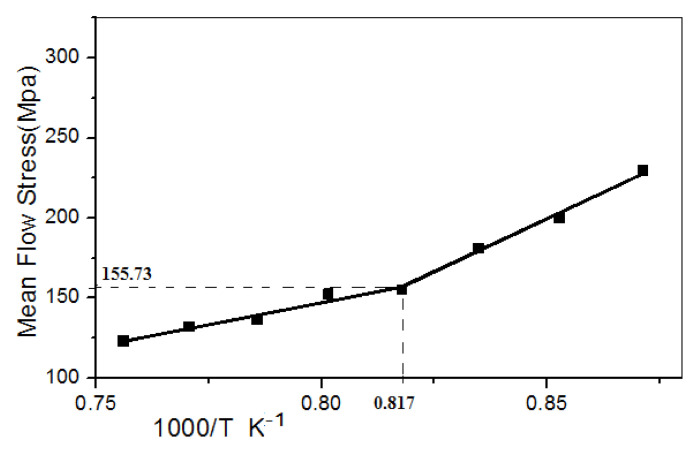
Graph of MFS against the inverse of absolute temperature.

The mean flow stress plot is depicted in Figure 7, with an increase in inverse absolute temperature. Figure 7 shows a discontinuity at flow mean stress (155.73 MPa) and inverse absolute temperature (0.817 K^−1^). This inverse absolute value gives non-recrystallization temperature (T_NR_). 

T_NR_ is identified as the temperature below which the strain is accumulated and leads to a sharp increase in the accrued mean flow stress. The non-recrystallization temperature can be precisely defined by assuming a linear dependence of mean flow stress and 1000/T.

(8) $$T_{\mathrm{NR}}=\frac{1000}{0.817},\;T_{\mathrm{NR}}=1224\;K,\;T_{\mathrm{NR}}=951\;{}^{\circ}C$$

This value corresponds to 951 °C (T_NR_). Plot before discontinuity shows a fully recrystallized. Additionally, after the discontinuity point, the plot shows nil recrystallization (mean non-recrystallization). Therefore, from the above observation, it is clear that rolling at T_NR_ will form partially recrystallized grains in pancaked shape in the microstructure of Nb-Microalloyed steel due to compression.

The effect of niobium on T_NR_ as a sample was observed for the same composition. For T_NR_ determination with the same method and corresponding parameters in which T_NR_ is perceived to be about 920 °C, by which we can conclude that Nb significantly enhances the T_NR_ value by almost 30 °C. 

### 3.4. Microstructure Evolution of Rolled MA Steel for T_NR_ Verification

**Figure 8:**
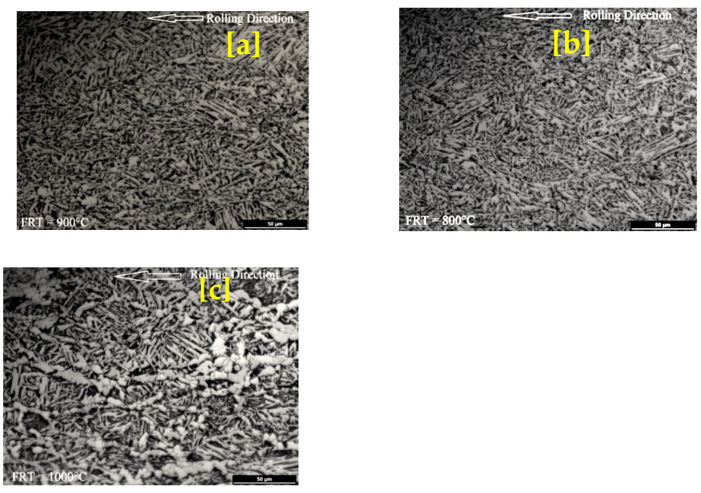
Optical micrographs of rolled niobium microalloyed steel in which finishing rolling temperature (**a**) 800; (**b**) 900; (**c**) 1000 °C.

**Figure 9:**
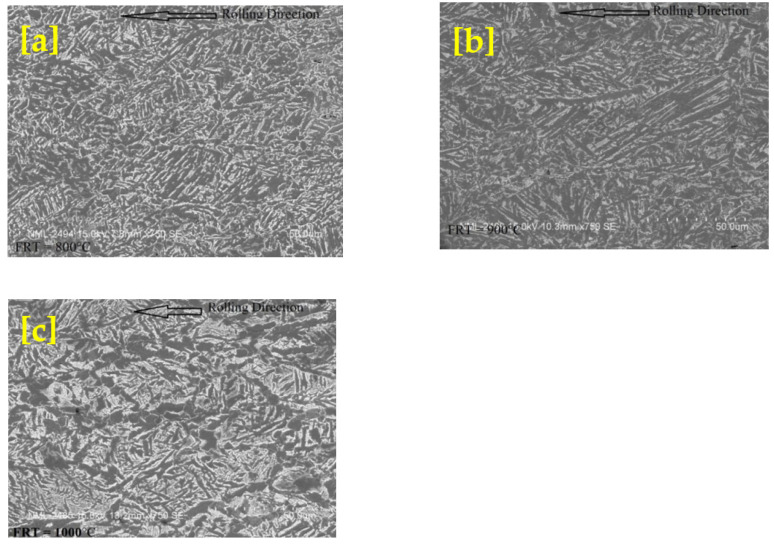
Scanning electron micrographs of rolled niobium microalloyed steel which finishing rolling temperature (**a**) 800; (**b**) 900; (**c**) 1000 °C.

Optical and scanning electron microstructures of rolled niobium microalloyed steel of finishing rolled temperature 800, 900, and 1000 °C are illustrated in Figure 8 and Figure 9. The microstructure shown in the figure consists of acicular ferrite (lathlike ferrite) + bainite/martensite in all rolled conditions. However, fineness decreases with increasing finished rolled temperature from 800 °C to 1000 °C. Bainite and acicular ferrite dominate in the microstructure, and there was no polygonal ferrite in Figure 8 and Figure 9. Figure 8c and Figure 9c show that the Nb-containing steel’s hot rolling refines the bainite and acicular ferrite. Polygonal ferrite is not observed in any rolled niobium microalloyed steel. The microstructure exhibited interlocking acicular ferrite morphology but possessed shorter laths. The T_NR_ of High-Nb steel alloy is found by the “mean flow stress” method, respectively, 951 °C. With observed T_NR_ it is clear that Nb’s use will enhance the T_NR_ value of a steel alloy, which makes it easy to improve strength by hot rolling at comparatively high temperatures. For the design of the rolling schedule, this temperature is considered. The finish rolling should be applied as much as possible under this temperature to achieve deformed austenite grains. This subsequently causes grain refinement after transformation. It is known in the industry that the region between T_NR_ and T_r_ has somewhat unpredictable recrystallization behavior. This results in an almost duplexlike microstructure of partially recrystallized grains and deformed grains. As a rule of thumb, deformation does not occur between the T_NR_ and T_r_, the region up to 50 °C–75 °C below T_NR_. The microstructure examined just above T_NR_ was about 50 °C above the T_NR_. A high fraction of recrystallized grains and microstructures below the T_NR_ were about 50 °C below the T_NR_. It shows no recrystallization, which means that T_NR_ is below 1000 °C and above 900 °C. Therefore, the microstructures confirmed the non-recrystallization temperature (T_NR_) range from 900 °C–1000 °C.

## 4. Conclusions

The purpose of this work was to determine the non-recrystallization temperature (T_NR_) in Nb-bearing microalloyed steel. In this investigation, the T_NR_ of Nb-bearing microalloyed steel was determined by multiple hit deformation tests in gleeble and confirmed with microstructure evolution. In this regard, the composition of Nb microalloyed steel was 0.23C-0.197Si-1.86Mn-0.0241P-0.008S-0.034Cr-0.005Mo-0.022Ni-0.013Cu-0.079Al-0.11Nb-0.004Ti -0.004N-Fe. The non-recrystallization temperature of given Nb-microalloyed steel was found at 951 °C for the aforementioned composition and its associated test parameters. It is worth noting that fully recrystallized grains were obtained at a finishing rolling temperature of 1000 °C, whereas no recrystallized grains were obtained at 900 °C. The microstructure consists of acicular ferrite + bainite/martensite in all rolling conditions. On the contrary, higher refined grains were observed at finishing a rolling temperature of 800 °C. Nonetheless, the formulated microstructure confirms the non-recrystallization temperature (T_NR_) which ranges between 900 °C–1000 °C. Moreover, there is room for researchers to develop specific approaches to predict the structural properties of these steels, which comprise numerous quantifiable microstructure features.

## Figures and Tables

**Table 1:** Chemical compositions of microalloyed steels (wt.%).

Material ID	MA Element (wt.%)	C	Si	Ti	P	N	Mn	Al	S	Fe
Base Alloy	Nil	0.2–0.22	0.2	0.004	0.03	0.006	1.5–2.0	0.03	0.001	Bal.
Low Nb	0.04–0.06	0.2–0.22	0.2	0.004	0.03	0.006	1.5–2.0	0.03	0.001	Bal.
High Nb	0.1–0.12	0.2-.22	0.2	0.004	0.03	0.006	1.5–2.0	0.03	0.001	Bal.

**Table 2:** Nb-MA steel composition in wt.%.

C	Mn	Cr	P	Cu	Si	Al	Ti	Mo	Ni	Nb	N
0.23	1.862	0.034	0.0241	0.013	0.197	0.0793	0.0042	0.005	0.022	0.11	0.004

**Table 3:** Quantitative results of the compression test in gleeble.

No. of Hit	Temp. of the Hit (°C)	The Area under the Curve (MJ/m^3^)	Strain/Hit	Mean Flow Stress (MPa)	1000/T (K^−1^)
1	1100	15.13	0.15	100	0.728332
2	1075	20.04	0.15	133	0.74184
3	1050	22.15	0.15	147	0.755858
4	1025	20.47	0.15	136	0.770416
5	1000	23.14	0.15	154	0.785546
6	975	22.04	0.15	146	0.801282
7	950	25.97	0.15	173	0.817661
8	925	28.76	0.15	191	0.834725
9	900	33.94	0.15	226.266	0.852515
10	875	35.48	0.15	236.533	0.87108

## Data Availability

The dataset can be requested from the corresponding authors upon a formal request.
